# Nuclear Phosphatidylinositol-Phosphate Type I Kinase α-Coupled Star-PAP Polyadenylation Regulates Cell Invasion

**DOI:** 10.1128/MCB.00457-17

**Published:** 2018-02-12

**Authors:** Sudheesh A.P., Rakesh S. Laishram

**Affiliations:** a Cardiovascular and Diabetes Biology Group, Rajiv Gandhi Centre for Biotechnology, Trivandrum, India

**Keywords:** 3′-end RNA processing, Star-PAP, PIPKIα, nuclear phosphoinositide signal, cell invasion, phosphorylation

## Abstract

Star-PAP, a nuclear phosphatidylinositol (PI) signal-regulated poly(A) polymerase (PAP), couples with type I PI phosphate kinase α (PIPKIα) and controls gene expression. We show that Star-PAP and PIPKIα together regulate 3′-end processing and expression of pre-mRNAs encoding key anti-invasive factors (*KISS1R*, *CDH1*, *NME1*, *CDH13*, *FEZ1*, and *WIF1*) in breast cancer. Consistently, the endogenous Star-PAP level is negatively correlated with the cellular invasiveness of breast cancer cells. While silencing Star-PAP or PIPKIα increases cellular invasiveness in low-invasiveness MCF7 cells, Star-PAP overexpression decreases invasiveness in highly invasive MDA-MB-231 cells in a cellular Star-PAP level-dependent manner. However, expression of the PIPKIα-noninteracting Star-PAP mutant or the phosphodeficient Star-PAP (S6A mutant) has no effect on cellular invasiveness. These results strongly indicate that PIPKIα interaction and Star-PAP S6 phosphorylation are required for Star-PAP-mediated regulation of cancer cell invasion and give specificity to target anti-invasive gene expression. Our study establishes Star-PAP–PIPKIα-mediated 3′-end processing as a key anti-invasive mechanism in breast cancer.

## INTRODUCTION

Pre-mRNA processing in the 3′ untranslated region (3′-UTR) is an essential step in eukaryotic gene expression that generates a polyadenosine tail at the mRNA 3′ end (polyadenylation) (1–3). A poly(A) tail is essential for the stability and translation efficiency of the mRNA (4, 5). All eukaryotic mRNAs except those encoding histones have a poly(A) tail at the 3′ end. 3′ polyadenylation is emerging as a crucial regulatory mechanism in various cellular functions and diseases (6, 7). Several key oncogenes and tumor suppressors are regulated through their 3′-UTRs (8–11). However, the role of pre-mRNA 3′-end processing in cell invasion and migration is still underexplored. Cellular invasiveness and migratory potential define the capability of cells to become motile and pilot through the extracellular matrix (ECM) within the tissue or to gain access to the adjacent tissues (12, 13). Cancer cells undergo migration and invasion that allow them to metastasize to other organs or tissues (14). Cells become invasive and migratory when they lose their attachment to the ECM and cell-cell junction proteins (12, 13). This transformation involves wide molecular changes and signaling events in the cell regulated through various mechanisms at the protein, DNA, and mRNA levels of various regulators and effector proteins (11, 15).

Poly(A) polymerases (PAPs) are enzymes responsible for the posttranscriptional 3′-end polyadenylation of pre-mRNAs (5, 16). Canonical PAPα/PAPγ are responsible for the polyadenylation of most nuclear mRNAs (5). The recent discovery of a variant PAP, Star-PAP (speckle-targeted PIPKIα-regulated PAP), indicates selective polyadenylation of nuclear mRNAs (17). Star-PAP targets distinct mRNAs involved in the oxidative-stress response, apoptosis, and cancer, and this targeting is independent of PAPα for the reported mRNAs (17–20). We have demonstrated that Star-PAP specificity for its target mRNAs is driven by a recognition sequence at the 3′-UTR, followed by exclusion of the canonical PAP via a suboptimal downstream sequence (DSE) located on its target mRNA UTR (21). While Star-PAP functions without certain cleavage factors, such as CstF-64, it requires additional associated proteins (17, 19, 21, 22). Moreover, as opposed to the canonical mechanism, Star-PAP directly binds the target mRNA and plays a structural role to help assemble the cleavage and polyadenylation complex (23).

Star-PAP activity is stimulated by the nuclear phosphoinositide second messenger phosphatidylinositol-4,5-bisphosphate (PI4,5P_2_), which in turn regulates Star-PAP target mRNA expression (17, 23). Microarray analysis after Star-PAP knockdown suggests that multiple cellular functions and signaling pathways are regulated by Star-PAP (17), yet the role of Star-PAP in cell invasion/migration is still undefined. Star-PAP is regulated by multiple signaling pathways, such as oxidative stress, DNA damage, and kinases (casein kinase Iα [CKIα]/CKIε and protein kinase Cδ [PKCδ]), and the nuclear phosphoinositide signal that regulates Star-PAP function (17, 19, 20, 22, 24). As the name suggests, Star-PAP interacts with and is regulated by the nuclear type I phosphatidylinositol (PI) phosphate kinase Iα (PIPKIα), which synthesizes nuclear PI4,5P_2_ (17, 20).

In the nucleus, there is an autonomous PI cycle distinct from that in the cytosol and involving similar sequential phosphorylation of the PI at 3, 4, or 5 hydroxy positions of the myoinositol ring by PI kinases into the inositol phosphate isomer PI4P or PI5P (25–28). PI4,5P_2_ is generated by phosphorylation of PIP isomers by PIP kinases (29, 30). Several nuclear PIPKs, such as PIPKIα, PIPKIγ, and PIPKIIβ, are now reported to target within the nucleus (25). PIPKIα is localized, along with Star-PAP, in the nuclear speckle that harbors factors for RNA processing/splicing and regulates 3′-end processing of mRNAs in key cellular functions (17, 26, 30, 31). Nuclear PI signaling is reported to regulate a vast array of functions, including gene expression; nuclear transport; actin polymerization; and chromatin remodeling, splicing, and mRNA processing (25, 27). PI4,5P_2_ controls various key effectors in cell-cell adhesion, cell migration and invasion, epithelial mesenchymal transition, and membrane transport (32, 33). However, the mechanistic role of nuclear PI4,5P_2_ or PIPKIα in the process of cell invasion/migration is unclear.

In this paper, we report novel nuclear PIPKIα-coupled 3′-end processing by the variant poly(A) polymerase Star-PAP, which regulates cell invasion in breast cancer cells. Star-PAP and PIPKIα together control a large subset of overlapping mRNA targets encoding anti-invasive factors. We observed different endogenous Star-PAP levels that correspond to variation in the invasiveness in different cell lines. Consistently, silencing of Star-PAP or PIPKIα individually or together increased cellular invasiveness in low-invasiveness MCF7 cells. Expression of increasing levels of Star-PAP resulted in a progressive decrease in cellular invasiveness in highly invasive MDA-MB-231 cells. Expression of PIPKIα-noninteracting Star-PAP mutants (ΔZF) and phosphodeficient (serine 6-to-alanine [S6A]) mutant Star-PAP in MDA-MB-231 cells did not have a significant effect on invasiveness, indicating the significance of Star-PAP–PIPKIα interaction and Star-PAP phosphorylation (serine 6) in regulation of cellular invasiveness. Our study establishes Star-PAP–PIPKIα-mediated 3′-end processing as a key anti-invasive regulatory mechanism in cancer metastasis.

## RESULTS

### Star-PAP and PIPKIα together control the expression and 3′-end processing of key anti-invasive regulators.

**FIG 1:**
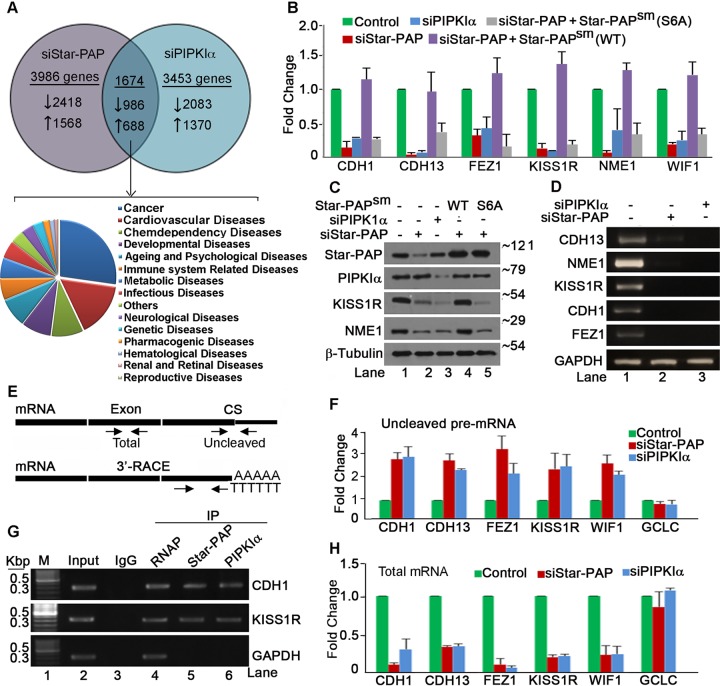
Star-PAP and PIPKIα together regulate expression and 3′-end processing of key anti-invasive factors. (A) Schematic showing the number of genes differentially regulated on knockdown of Star-PAP and PIPKIα and genome-wide pathway/functional analysis of Star-PAP- and PIPKIα-regulated genes. (A list of common genes significantly regulated by both Star-PAP and PIPKIα is provided in Table S1 in the supplemental material.) (B) qRT-PCR analysis of mRNAs encoding anti-invasive factors (*CDH1*, *CDH13*, *FEZ1*, *KISS1R*, *NME1*, and *WIF1*) with total RNA isolated from HEK 293 cells with Star-PAP and PIPKIα knockdown, and rescue with exogenous WT and S6A Star-PAP expression after silencing endogenous Star-PAP in HEK 293 cells, as indicated. The error bars indicate standard errors of the mean (SEM). (C) Western blot analysis of Star-PAP, PIPKIα, and target proteins as in panel B. Numbers on the right indicate molecular mass in kilodaltons. (D) 3′-RACE assay of *CDH13*, *NME1*, *KISS1R*, *CDH1*, *FEZ1*, and *GAPDH* from total RNA isolated from HEK 293 cells after Star-PAP or PIPKIα knockdown. − siRNA indicates that control scrambled siRNA was used. (E) Schematics of 3′-RACE (bottom) and cleavage (top) assays. CS, cleavage site. (F and H) Measurement of uncleaved pre-mRNA levels (F) expressed relative to total mRNA (H) after Star-PAP and PIPKIα knockdowns, as indicated. (G) RIP analysis of *CDH1* and *KISS1R* mRNAs after immunoprecipitation (IP) with antibodies specific to Star-PAP, PIPKIα, and control RNA Pol II and IgG from HEK 293 cells. Input, 10% of the IP lysate. M, marker lane.

Previous studies reported specific sets of mRNAs regulated by both Star-PAP and PIPKIα (17). We previously showed the direct interaction of Star-PAP with PIPKIα and that it occurred in the ZF region at the Star-PAP N terminus (20). However, this interaction needed phosphorylation of the serine 6 residue at the Star-PAP N terminus *in vivo*. Serine 6-to-alanine (S6A) mutation abolished the association between Star-PAP and PIPKIα (20). Analysis of Star-PAP- and PIPKIα-controlled mRNAs in HEK 293 cells (17) indicated that >40% of Star-PAP targets overlapped those of PIPKIα (Fig. 1A). Of these, >900 mRNAs were downregulated and >650 genes were upregulated in common in both Star-PAP and PIPKIα knockdown cells. The majority of these overlapping mRNAs (∼30%) were involved in various cellular events in cancer (Fig. 1A). Within the set of Star-PAP and PIPKIα targets, several genes that promote cancer were upregulated while several key antimetastatic regulators showed downregulation (see Table S1 in the supplemental material). The downregulated mRNAs included those encoding key anti-invasive regulators in breast cancer, such as E-cadherin (CDH1), metastatin/kissipetin receptor (KISS1R), cadherin-13 (CDH13), nucleoside diphosphate kinase A (NME1/NME23A), WNT inhibitory factor 1 (WIF1), and fasciculation and elongation protein zeta 1 (FEZ1) (34–36).

To investigate Star-PAP-mediated mRNA regulation, we knocked down Star-PAP and PIPKIα in HEK 293 cells (Fig. 1C) and analyzed the mRNA and protein expression of these factors by quantitative real-time PCR (qRT-PCR) and Western blotting. While we observed downregulation of protein (KISS1R and NME1) and mRNA (*CDH1*, *CDH13*, *FEZ1*, *KISS1R*, *NME1*, and *WIF1*) levels on either Star-PAP or PIPKIα knockdown, there was no effect on control *GCLC* and β-tubulin expression (Fig. 1B, C, and H). The loss of mRNA expression upon Star-PAP knockdown was rescued by the ectopic expression of FLAG-Star-PAP (Star-PAP^sm^) with silent mutations that rendered our small interfering RNA (siRNA)/short hairpin RNA (shRNA) ineffective (19) (Fig. 1B). However, the reduced mRNA expression was not rescued by PIPKIα-noninteracting Star-PAP (S6A mutant) (20) (Fig. 1B), indicating that Star-PAP–PIPKIα interaction and serine 6 phosphorylation are required for the regulation of target mRNA expression. Consistent results were observed in Western blot analysis of KISS1R and NME1 protein levels (Fig. 1C). These results indicate that PIPKIα interaction determines Star-PAP specificity for target mRNAs encoding anti-invasive regulators.

Further, 3′-end processing was analyzed by 3′-rapid amplification of cDNA ends (RACE) assay (23) and by measuring the cleavage efficiency of pre-mRNA 3′-UTRs (17) (Fig. 1E) after knockdown of Star-PAP or PIPKIα. We observed compromised 3′-end polyadenylation, as was evident from the reduced 3′-RACE products of *CDH1*, *NME1*, *KISS1R*, *CDH13*, and *FEZ1* with knockdown of either Star-PAP or PIPKIα (Fig. 1D). We then measured cleavage efficiency using a pair of primers across the cleavage site (21) (Fig. 1E). We detected accumulation of uncleaved pre-mRNA, while there was a reduction in the total mRNA level (Fig. 1F and H) similar to that with control CPSF-160 knockdown (see Fig. S1A and B and S3K in the supplemental material). Moreover, treatment with actinomycin D or knockdown of a control *CDH1* transcriptional regulator, FOXA2 (see Fig. S3L in the supplemental material), did not affect the uncleaved RNA fractions of *CDH1* (actinomycin D treatment and FOXA2 knockdown) and *CDH13* (actinomycin D treatment) mRNAs despite a reduction in the total mRNA (see Fig. S1A and B in the supplemental material). Consistently, treatment with a polyadenylation inhibitor, cordycepin, did not show any effect on cleavage efficiency but reduced the total mRNA level (see Fig. S1A and B in the supplemental material). These results indicate that the loss of mRNA expression on Star-PAP or PIPKIα knockdown is a result of compromised cleavage at the 3′-UTRs of target pre-mRNAs. RNA immunoprecipitation (RIP) analysis showed association of both Star-PAP and PIPKIα on UTRs of *CDH1* and *KISS1R*, while there was no association on nontargets, such as the *GAPDH* (glyceraldehyde-3-phosphate dehydrogenase) mRNA UTR (Fig. 1G). Interestingly, Star-PAP S6A associated with target mRNAs (albeit weakly) (20) but did not rescue the expression of Star-PAP target mRNAs (Fig. 1B). Together, these results demonstrate that Star-PAP and PIPKIα regulate the 3′-end processing of key anti-invasive factors, such as CDH1, NME1, CDH13, FEZ1, WIF1, and KISS1R.

### Star-PAP expression negatively regulates cellular invasiveness in breast cancer cells.

**FIG 2:**
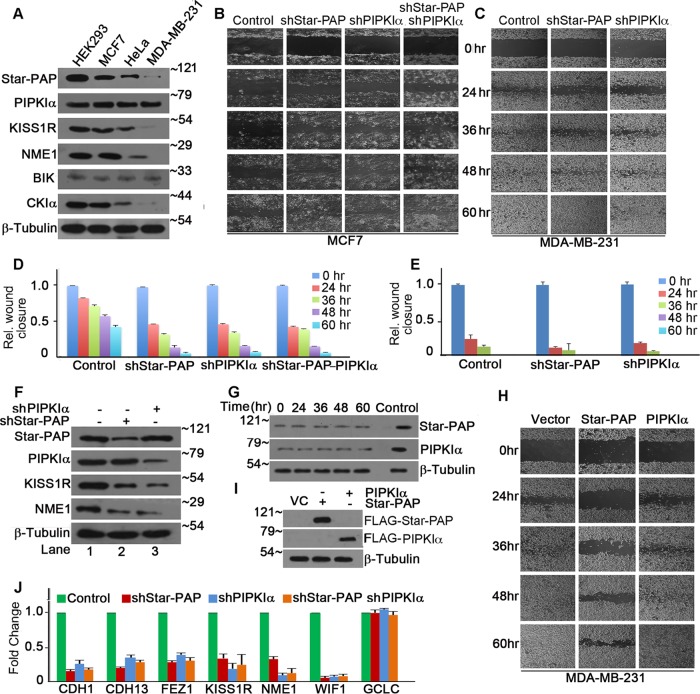
Star-PAP negatively regulates the invasive potential of breast cancer cells. (A) Western blot showing endogenous levels of Star-PAP and PIPKIα, along with Star-PAP targets KISS1R, NME1, and BIK and loading control β-tubulin from HEK 293, MCF7, HeLa, and MDA-MB-231 cells, as indicated. Numbers on the right indicate molecular mass in kilodaltons. (Quantification of the blots is shown in Fig. S1C to E in the supplemental material.) (B) Analysis of wound closure on collagen-treated plates after blocking cell proliferation with mitomycin C at various time points (0 to 60 h) postscratch in MCF7 cells after lentivirus-based stable knockdown of Star-PAP and PIPKIα and combined knockdown of both, as indicated (representative of 3 independent experiments). (Protein levels of Star-PAP and PIPKIα at various time points are shown in panel G.) (C) Wound healing assay, as in panel B, of MDA-MB-231 cells after Star-PAP or PIPKIα knockdown, as indicated. (D) Quantification of wound closure in panel B expressed relative to that at time zero postscratch. (The actual measurements of wound gaps [in micrometers] are plotted in Fig. S4A in the supplemental material.) The error bars represent SEM of 3 independent experiments. (E) Quantification of wound closure in panel C expressed relative to that at time zero. (The actual wound gaps are plotted in Fig. S4B in the supplemental material.) The error bars represent SEM of 3 independent experiments. (F) Western blot of Star-PAP, PIPKIα, CKIα, and Star-PAP targets KISS1R and NME1 from MCF7 cell lysates after Star-PAP or PIPKIα knockdown. − shRNA indicates that control shRNA was used. Numbers on the right indicate molecular mass in kilodaltons. (G) Western blots of Star-PAP and PIPKIα at various time points postscratch (in panel B). Numbers on the left indicate molecular mass in kilodaltons. (H) Wound healing assay as in panel C with ectopic expression of FLAG epitope-tagged Star-PAP^sm^ and -PIPKIα in MDA-MB-231 cells. (Quantification of wound closure relative to that at time zero postwounding and actual wound gaps [in micrometers] are shown in Fig. S1G and S4C in the supplemental material, respectively.) (I) Western blot analysis of FLAG-Star-PAP and -PIPKIα after overexpression in MDA-MB-231 cells. Numbers on the left indicate molecular mass in kilodaltons. VC, vector control. (J) qRT-PCR analysis of various Star-PAP targets, as indicated, after individual and combined knockdown of Star-PAP and PIPKIα (*n* = 3). The error bars represent SEM.

To investigate the role of Star-PAP in cellular invasiveness, we examined the levels of Star-PAP in various cancer cell lines (MCF7, MDA-MB-231, HeLa, and control HEK 293 cells). MCF7, a breast cancer cell line with low invasiveness, expressed high endogenous levels of Star-PAP, while the highly invasive MDA-MB-231 cells expressed negligible levels of Star-PAP protein (Fig. 2A; see Fig. S1C in the supplemental material). HeLa cells expressed Star-PAP at intermediate levels, while HEK 293 cells expressed high Star-PAP levels (Fig. 2A) compared to MDA-MB-231 and MCF7 cells. The endogenous Star-PAP levels in various cell lines were inversely related to the invasiveness of the cell line (see Fig. S1F and H in the supplemental material). However, there was no significant difference in PIPKIα levels in these cell types (Fig. 2A; see Fig. S1D in the supplemental material). Star-PAP targets, such as KISS1R or NME1, also exhibited different expression patterns in different cell lines proportionate to the relative Star-PAP levels (Fig. 2A; see Fig. S1E in the supplemental material). Interestingly, another Star-PAP target, Bcl2-interacting killer (BIK) protein (19), did not exhibit significant differences among the different cell lines (Fig. 2A).

To further study the role of Star-PAP in cellular invasiveness, we knocked down Star-PAP in MCF7 cells using a stable lentiviral (shStar-PAP) system (Fig. 2F) and, in parallel, ectopically expressed Star-PAP^sm^ in MDA-MB-231 cells (Fig. 2I). We then tested for invasiveness by transwell invasion assays and wound healing assays (37–39) on collagen-coated plates after blocking cell proliferation with mitomycin C treatment (Fig. 2B to E and H; see Fig. S2E to H in the supplemental material). While only ∼55% wound closure was observed 60 h postscratch in normal MCF7 cells, Star-PAP knockdown resulted in similar healing in <24 h postscratch, and wounds were almost completely healed by 48 h postscratch (Fig. 2B and D; see Fig. S4A in the supplemental material). PIPKIα knockdown (Fig. 2F; see Fig. S3E in the supplemental material), too, resulted in a similar enhancement in healing (∼36-h improvement) (Fig. 2B and D; see Fig. S4A in the supplemental material). There was no significant difference in wound healing times upon Star-PAP–PIPKIα combined knockdown from that after individual knockdowns, suggesting roles of both Star-PAP and PIPKIα in the process. Star-PAP and PIPKIα protein levels at various time points (0 to 60 h) postscratch during wound healing are shown in Fig. 1G. Star-PAP or PIPKIα knockdown (see Fig. S3G and H in the supplemental material) in control HeLa cells also resulted in a similar trend of accelerated wound healing (see Fig. S2A to C in the supplemental material). Similar results were obtained in transwell invasion assays (see Fig. S2E to H in the supplemental material). There was a >30% increase in invasion upon knockdown of either Star-PAP or PIPKIα toward the lower chamber compared to that in control cells at 48 h (see Fig. S2E and G in the supplemental material).

Consistent with the low expression level in MDA-MB-231 cells, Star-PAP knockdown (see Fig. S3J in the supplemental material) did not have a marked effect on wound closure in the wound healing assay (Fig. 2C and E; see Fig. S4B in the supplemental material). Although high endogenous PIPKIα levels were observed in MDA-MB-231 cells (where the Star-PAP level is minimal), PIPKIα knockdown (see Fig. S3I in the supplemental material) did not have a significant effect on wound closure (Fig. 2C and E), indicating the involvement of a close nexus between Star-PAP and PIPKIα in regulating cellular invasiveness. Ectopic expression of Star-PAP in MDA-MB-231 cells delayed the wound healing process (Fig. 2I and H; see Fig. S1G and S4C in the supplemental material). While normal cells recovered at ∼24 h, Star-PAP-overexpressing cells required >60 h for total wound closure. PIPKIα overexpression had a negligible effect on the healing process (Fig. 2H and I; see Fig. S1G and S4C in the supplemental material), consistent with its invariant level in different cell types. Similarly, in the transwell invasion assay, we observed a >25% decrease in invasion toward the lower chamber upon ectopic Star-PAP expression compared to that with the vector control (see Fig. S2F and H in the supplemental material).

Consequently, there was reduced mRNA expression of target anti-invasive factors, such as *CDH1*, *NME1*, *CDH13*, *FEZ1*, *KISS1R*, and *WIF1*, upon Star-PAP or PIPKIα knockdown in MCF7 cells (Fig. 2J; see Fig. S3E and F in the supplemental material). As observed in HEK 293 cells, double knockdowns and individual knockdowns of Star-PAP and PIPKIα exhibited similar levels of reduction of target mRNAs in MCF7 cells (Fig. 2J). Moreover, Western blot analysis also showed reduced expression of target proteins (KISS1R and NME1) upon Star-PAP and PIPKIα knockdown (Fig. 2F). Similarly, ectopic Star-PAP overexpression resulted in 4- to 6-fold induction of mRNA levels of the target mRNAs *CDH13*, *NME1*, *KISS1R*, and *FEZ1* in MDA-MB-231 cells, which are otherwise marginally expressed in these cells (see Fig. S2D in the supplemental material). These results confirm that Star-PAP regulates cellular invasiveness by controlling the expression of key anti-invasive factors in breast cancer cells.

### Cellular invasiveness is inversely correlated with ectopically expressed Star-PAP protein levels in MDA-MB-231 cells.

**FIG 3:**
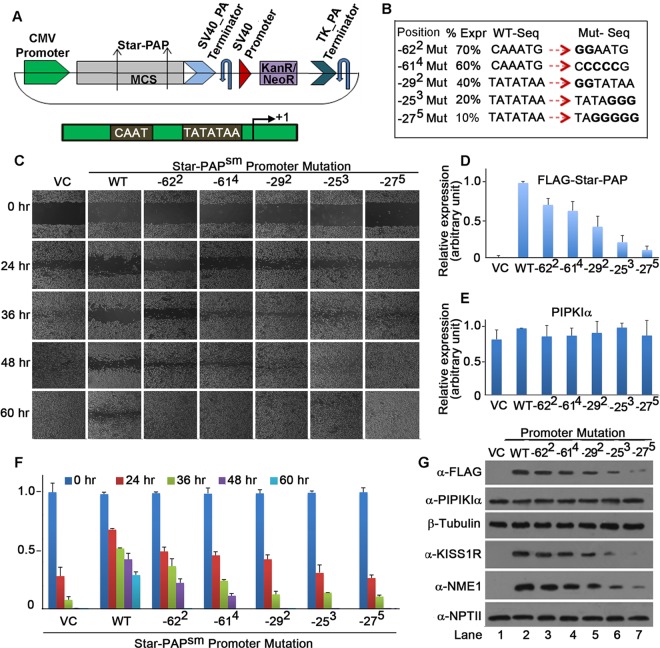
Increasing expression of Star-PAP results in a relative decrease in cellular invasiveness. (A) Schematic showing the FLAG-tagged Star-PAP construct under the control of the CMV promoter (the CAAT box and TATATAA box are shown below). (B) Tabular representation of wild-type CMV promoter CAAT and TATAAT sequences and various mutations generated by site-directed mutagenesis. The position of the mutation with respect to the +1 start site is indicated (the superscript number indicates the number of nucleotides changed). Expr, expression. (C) Wound healing assay, as in Fig. 2C, after ectopic expression of various promoter mutant Star-PAP constructs in MDA-MB-231 cells after knockdown of Star-PAP (the image is representative of 3 independent experiments). (D and E) Quantification of Star-PAP (D) and PIPKIα (E). The error bars represent SEM (*n* = 3). (F) Quantification (wound closure relative to that at time zero postwounding) of the wound healing (in panel C) in 3 independent experiments. The error bars represent SEM. (The actual wound gap at each time point is shown in Fig. S4E in the supplemental material.) (G) Western blot showing FLAG-Star-PAP levels expressed from various promoter mutations (shown in panel B) after transient expression in MDA-MB-231 cells. Control PIPKIα, loading control β-tubulin, Star-PAP targets KISS1R and NME1, and neomycin phosphotransferase II expressed from an independent promoter in the same vector as Star-PAP are shown.

To further test the role of Star-PAP in cellular invasiveness, we generated several cytomegalovirus (CMV) promoter mutations on the Star-PAP construct (Fig. 3A) to express different levels of Star-PAP, as reported previously (40), in MDA-MB-231 cells (Fig. 3B; see Fig. S3A in the supplemental material). While mutations of the CAAT box (−62 and −61) resulted in 70% and 60% Star-PAP expression, respectively, mutations of the TAATA box (−29, −25, and −27) resulted in 40%, 20%, and 10% Star-PAP expression levels, respectively, relative to wild-type (WT) expression (taken as 100%) (Fig. 3B, D, and G). Expression of these mutants in MDA-MB-231 cells resulted in a progressive decline in the time taken for wound closure in a manner inversely proportional to Star-PAP levels in the cell (Fig. 3C). While wild-type Star-PAP expression required >60 h for wound closure, ∼10% Star-PAP expression (−27) resulted in wound closure at ∼24 h, similar to what occurred with the pCMV vector control (Fig. 3C and F). Similarly, other mutants followed this increasing order of invasiveness (WT < −62 < −61 < −29 < −25 < −27 mutations) (Fig. 3C and F; see Fig. S4E in the supplemental material). We also observed decreased expression of the Star-PAP targets NME1 and KISS1R with decreasing Star-PAP levels (Fig. 3G; see Fig. S3B and C in the supplemental material). There was no difference in PIPKIα levels and neomycin phosphotransferase II (NPTII), an expression/transfection control expressed from an independent promoter in the same Star-PAP plasmid construct (Fig. 3E and G; see Fig. S3D in the supplemental material). This confirms the negative role of Star-PAP in cellular invasiveness that operates via regulation of its target mRNAs.

### Star-PAP requires PIPKIα interaction and S6 phosphorylation to regulate cellular invasiveness.

**FIG 4:**
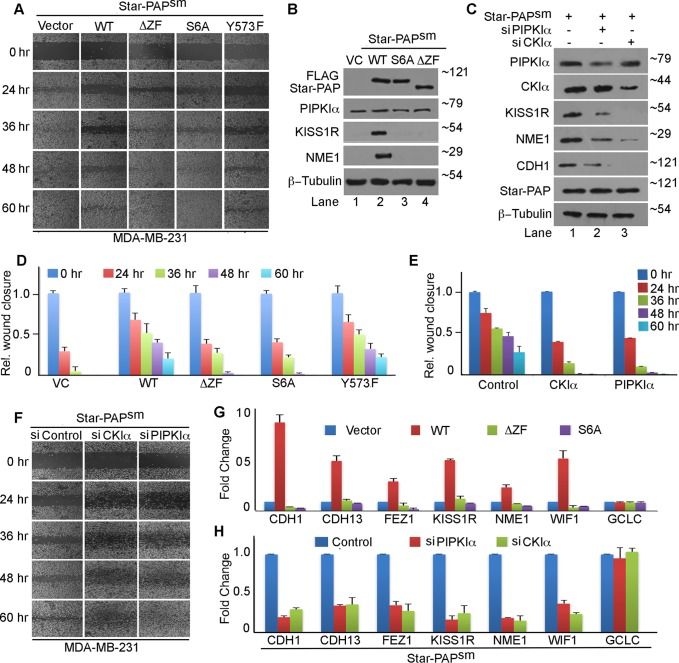
Star-PAP–PIPKIα interaction and Star-PAP serine 6 phosphorylation regulate cellular invasiveness. (A) Wound healing assay (as in Fig. 2H) in the presence of ectopic expression of Star-PAP deletion and point mutations (ZF deletion and S6A and Y573F mutant Star-PAP) after endogenous Star-PAP knockdown. (B) Western blot analysis of WT Star-PAP and various mutants, as in panel A. Star-PAP coregulator PIPKIα, loading control β-tubulin, and Star-PAP targets KISS1R and NME1 are indicated. Numbers on the right indicate molecular mass in kilodaltons. (C) Western blot analysis of Star-PAP; PIPKIα; Star-PAP targets NME1, KISS1R, and CDH1; control β-tubulin; and CKIα from MDA-MB-231 cells after knockdown of PIPKIα and CKIα in the presence of ectopic FLAG-Star-PAP expression. Numbers on the right indicate molecular mass in kilodaltons. (D) Quantification of wound closure at various time points in panel A expressed relative to the 0-h time point postscratch. The error bars represent SEM (*n* = 3). (A plot of the actual wound gaps at various time points is shown in Fig. S4D in the supplemental material.) (E) Quantification of wound closure in panel F expressed relative to the 0-h time point. The error bars represent SEM (*n* = 3). (A plot of the actual wound gaps at various time points is provided in Fig. S4F in the supplemental material.) (F) Wound healing assay as in panel A but after knockdown of PIPKIα or CKIα in the presence of ectopically expressed wild-type Star-PAP^sm^. (Quantifications are shown in panel E and Fig. S4F in the supplemental material; *n* = 3.) (G) qRT-PCR analysis of various Star-PAP targets, as indicated, under conditions similar to those in panel B. (H) qRT-PCR analysis of various Star-PAP targets, as indicated, under conditions similar to those in panel C.

Star-PAP requires PIPKIα to regulate its subset of overlapping target mRNAs in HEK 293 cells. Therefore, we tested cellular invasiveness in MDA-MB-231 cells after expression of the PIPKIα-noninteracting Star-PAP (Fig. 4B). While wild-type Star-PAP decreased the time required for wound healing, the ZF deletion (PIPKIα interaction domain) Star-PAP did not have any effect on the healing process (Fig. 4A and D; see Fig. S4D in the supplemental material). A serine 6-to-alanine (S6A) mutation that is deficient in S6 phosphorylation (required for *in vivo* PIPKIα association and Star-PAP RNA binding) also showed effects similar to those with the ZF deletion (Fig. 4A and D), suggesting a role for Star-PAP–PIPKIα interaction and Star-PAP phosphorylation in the wound healing process. Similarly, in transwell invasion assays, wild-type Star-PAP, but not S6A Star-PAP, showed reduction in invasiveness (see Fig. S2F and H in the supplemental material). Mutation of yet another control putative phosphorylation site on Star-PAP (Y573F) did not have any effect on wound closure (Fig. 4A and D). The marginal expression of Star-PAP target proteins (KISS1R and NME1) was rescued by wild-type Star-PAP expression but not by ZF deletion or S6A mutant Star-PAP expression (Fig. 4B). qRT-PCR analysis also showed induced mRNA levels of the Star-PAP targets *CDH1*, *CDH13*, *KISS1R*, *NME1*, and *WIF1* on wild-type Star-PAP expression and not with either the ZF deletion or S6A mutant Star-PAP expression in MDA-MB-231 cells (Fig. 4G).

To further confirm the requirement for PIPKIα, we knocked down PIPKIα in the presence of ectopically expressed wild-type Star-PAP in MDA-MB-231 cells (Fig. 4C). We observed no effect of the ectopic Star-PAP expression on wound closure when PIPKIα was depleted in the cells (Fig. 4F; see Fig. S4F in the supplemental material). Since CKIα phosphorylates S6 on the ZF domain in Star-PAP (20), we knocked down CKIα (Fig. 4C) and tested the effect on wound closure. As expected, the effect of ectopic Star-PAP expression was diminished on CKIα knockdown (Fig. 4F; see Fig. S4F in the supplemental material), consistent with PIPKIα knockdown, indicating the requirement for S6 phosphorylation in the wound healing process. Expression of the targets CDH1, KISS1R, and NME1 was also reduced on CKIα knockdown despite the exogenous expression of Star-PAP (Fig. 4C). Similar results were obtained in qRT-PCR analysis, as well, where mRNA expression (*CDH1*, *NME1*, *CDH13*, *KISSIR*, *WIF1*, *FEZ1*, and *CDH13*) was reduced even when ectopic Star-PAP was expressed in MDA-MB-231 cells (Fig. 4H). These results reveal that S6 Star-PAP phosphorylation and the Star-PAP–PIPKIα interaction are crucial for the regulation of cancer cell invasion.

## DISCUSSION

**FIG 5:**
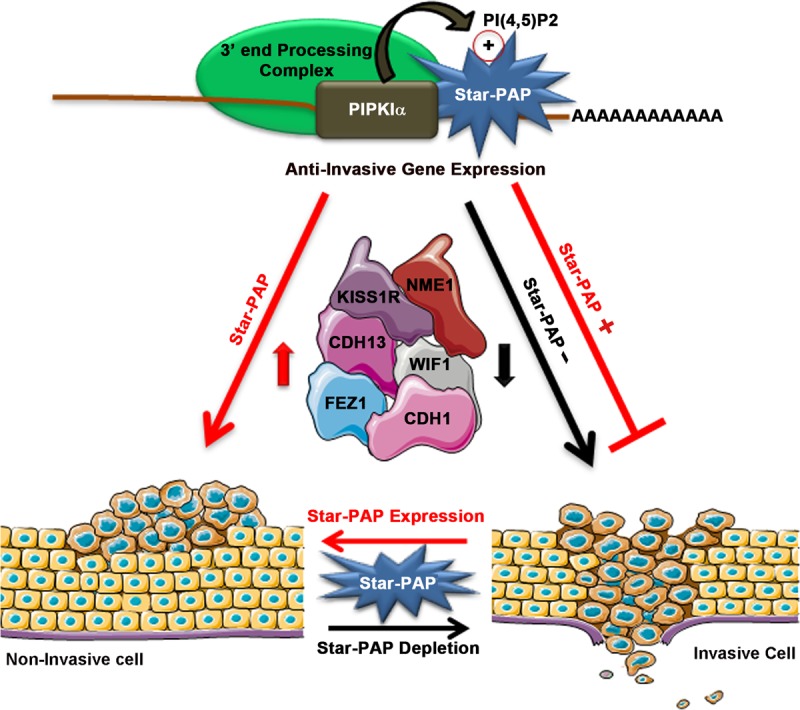
Model of Star-PAP–PIPKIα interaction-mediated regulation of cancer cell invasion.

Phosphoinositide signaling in cancer is associated primarily with the oncogenic PI3K/Akt/mTORC1 cascade (41–43). Recent studies suggest that the lipid messenger PI4,5P_2_ is not merely a substrate for phosphatidylinositol (3,4,5)-triphosphate (PI3,4,5P_3_) generation but is also a key regulator of various cellular events in cancer (44). PI4,5P_2_ and its synthesizing enzymes, the PIP kinases, regulate cellular motility, polarity, and invasion, which are integral to cancer progression (44, 45). However, this regulation is mostly limited to cytosolic PI4,5P_2_, and the importance of its nuclear counterpart in cell invasion or metastasis is largely undefined (25, 44). The type I PIP kinase α is a key PI4,5P_2_-generating enzyme inside the nucleus that is speckle localized, along with the noncanonical PAP Star-PAP (25, 27). Our work demonstrates a novel role of nuclear PIPKIα in cellular invasiveness in breast cancer cells by coupling with Star-PAP-mediated 3′-end processing to control the expression of important anti-invasive regulators, such as CDH1, KISS1R, NME1, CDH13, WIF1, and FEZ1 (35, 36). Concurrently, the Star-PAP–PIPKIα complex was observed to elicit an anti-invasive property in the cell. Both proteins are required in this process, and the absence of either Star-PAP or PIPKIα resulted in loss of regulation. A model for PIPKIα-coupled Star-PAP regulation of cellular invasiveness is shown in Fig. 5.

Cellular invasiveness is directly linked to several innate functions of a cell, such as immune response, development, wound healing, cancer progression, and metastasis (13). Cancer cells progress from migration and invasion and metastasize to neighboring tissues to form secondary tumors (13). We have established Star-PAP as a negative regulator of cellular invasiveness in breast cancer cells, indicating a putative role of Star-PAP in preventing cancer metastasis. A recent study has shown a role of Star-PAP as a tumor suppressor that prevents cell proliferation by a yet undefined mechanism (46). Star-PAP antitumor activity was implicated through a previously identified Star-PAP target, the apoptotic BIK gene (19). This is not the case, however, in cellular invasion, as there was no difference in the levels of BIK protein among different cancer cell lines, while Star-PAP levels differed and corresponded to cellular invasiveness. Moreover, BIK expression is independent of CKIα (19), which is involved in the regulation of cellular invasiveness. Knockdown of PKCδ, which regulates BIK expression through Star-PAP (19), also has no effect on cellular invasiveness (data not shown). This indicates a distinct pathway for metastasis, independent of the reported BIK-mediated apoptotic or tumor-suppressive function. We have shown a role for Star-PAP in cellular invasiveness occurring via control of several critical anti-invasive/metastatic factors in the cell.

The mechanism by which Star-PAP selects target mRNAs involved in the oxidative-stress response and apoptosis has been reported (19, 21, 23). Star-PAP recognizes an AUA-containing motif flanked by GC-rich elements. A suboptimal downstream sequence (DSE) on Star-PAP target UTRs excludes recruitment of canonical PAPα via CstF-64 (21). There are similar signature sequence elements on the UTRs of anti-invasive factors such as CDH1, NME1, or KISS1R (data not shown). This raises important questions: is sequence-driven specificity sufficient to regulate multiple cellular functions via Star-PAP, and what drives specificity for mRNAs in cellular invasiveness? Our data indicate that this specificity is driven by the interaction with PIPKIα for this set of overlapping target mRNAs. This observation is consistent with our previous report that the Star-PAP–mRNA interaction is modulated by PI4,5P_2_ binding *in vitro* (20). On the other hand, there are Star-PAP targets that are independent of PIPKIα (17, 20), suggesting involvement of other coregulators in controlling different cellular functions. We have identified another unique Star-PAP coregulator RNA binding protein (RBM10) that functions with Star-PAP, and together they regulate cardiac hypertrophy (unpublished data). Star-PAP also associates with the kinases CKIα/CKIε and PKCδ, but they regulate distinct mRNA targets (19, 22). For example, while CKIα association with Star-PAP is involved in the oxidative-stress response, PKCδ association regulates the DNA damage pathway (19).

The Star-PAP–PIPKIα nexus in cellular invasiveness is largely similar to that of the stress response pathway in terms of its regulation. Both require CKIα-mediated phosphorylation at S6 in the ZF motif. Interestingly, S6 phosphorylation is required for Star-PAP nuclear retention, yet it is selective in regulation of Star-PAP target mRNAs (20). It appears to function in parallel with PIPKIα (20) to regulate cellular invasiveness or cancer. Currently, it is unclear if Star-PAP requires additional phosphorylation(s) to regulate cellular invasiveness. Nevertheless, our results suggest that phosphorylation(s) on Star-PAP regulates different cellular functions. While the serine 6 phosphorylation site for CKIα is known, PKCδ-targeted sites are still unidentified (19, 20). Moreover, reports indicate that there are additional protein kinases associated with Star-PAP, consistent with its role in Star-PAP function (22, 24). However, it is still unclear if all functional Star-PAP molecules in the nucleus are phosphorylated. Further experiments will reveal definitive answers to these questions. Nonetheless, our preliminary data largely suggest that differentially phosphorylated Star-PAP is involved in signaling regulation and cellular functions, such as invasion, *in vivo* (data not shown). Nonetheless, Escherichia coli purified recombinant Star-PAP shows polyadenylation activity *in vitro* (17). This suggests that Star-PAP phosphorylation is largely signal driven and that its cellular functions are coupled to coregulators such as PIPKIα.

## MATERIALS AND METHODS

### Cell culture and transfections.

Human embryonic kidney (HEK 293) cells, HeLa cell lines, MCF7 (estrogen receptor (ER)/progesterone receptor (PR)-positive human breast cancer) cells, and MDA-MB-231 (triple-negative human breast cancer) cells were obtained from the American Type Culture Collection and maintained in Dulbecco's modified Eagle's medium (DMEM) with 10% fetal bovine serum (FBS) and penicillin-streptomycin (50 U/ml) at 37°C in 5% CO_2_. Plasmid transfections were carried out using the calcium phosphate method (HEK 293 and HeLa cells) and Lipofectamine (Invitrogen) (MCF7 and MDA-MB-231 cells) or by siRNA using Oligofectamine (Invitrogen) according to the manufacturer's instructions. Cells were harvested 48 h posttransfection. The RNA oligonucleotides used for knockdown are shown in the supplemental material. A lentiviral vector system, pLKO.1-TRC, was modified to express an shRNA specific to Star-PAP (shStar-PAP) or PIPKIα (shPIPKIα) under the U6 promoter as described previously (47). Lentiviral particles were generated in HEK 293 cells as described previously (48). Stable knockdown cells were selected using 10 μg/ml of puromycin (Sigma) (49).

### Immunoblotting.

Immunoblotting experiments were carried out using appropriate antibodies as described previously (20). The intensities of bands were quantified with ImageJ software. A list of antibodies is provided in the supplemental material.

### 3′-RACE assay and cleavage measurement.

Total RNA was isolated from HEK 293 cells using an RNeasy mini kit (Qiagen) as described previously (21). 3′-RACE assays were carried out using the 3′-RACE system (Invitrogen) according to the manufacturer's instructions with 2 μg of total RNA as described previously (21). The RACE products were confirmed by sequencing. For measurement of cleavage efficacy, uncleaved mRNA levels were measured by qRT-PCR using a pair of primers across the cleavage site as described previously (17). The noncleaved messages were expressed as a ratio over the total mRNA, and all samples (siRNA Star-PAP or PIPKIα knockdowns) were expressed as the fold change from the control for the ratio. The gene-specific primers used in 3′-RACE and cleavage assays are provided in the supplemental material.

### RIP.

RNA immunoprecipitation experiments were carried out after cross-linking proteins and RNA with 1% formaldehyde in HEK 293 cells using specific antibodies against Star-PAP, PIPKIα, and RNA polymerase II (Pol II), as described previously (21, 50). Briefly, we cross-linked total cellular proteins with nucleic acids in the cell with formaldehyde. Nuclear fractions were then isolated, followed by shearing of the nucleic acids by sonication into smaller fragments (∼500 nucleotides [nt]), as described previously (21, 50). The protein bound to the RNA was immunoprecipitated using specific antibodies. The immunoprecipitated samples were eluted, de-cross-linked, and digested with DNase I to remove the associated DNA. The associated RNA was then detected using RT-PCR with gene-specific primers. The gene-specific primers used for detecting *CDH1*, *KISS1R*, and *GAPDH* are listed in the supplemental material.

### qRT-PCR.

qRT-PCR was carried out as described previously (20) in a CFX98 multicolor system (Bio-Rad) using 2 μg of total RNA reverse transcribed with an RT-PCR kit (Bio-Rad). Single-product amplification was confirmed by melting-curve analysis, and primer efficiency was near 100% in all experiments. Quantifications are expressed in arbitrary units, and target mRNA abundance was normalized to the expression of GAPDH by the method of Pfaffl (52). All the qRT-PCR results are representative of at least three independent experiments (*n* > 3). A list of primers is provided in the supplemental material.

### Invasion assays.

Invasion assays were carried out using scratch healing and transwell invasion assays, as described previously (37–39). Briefly, monolayers of cells with knockdowns or transfections were grown on culture plates coated with extracellular matrix (collagen). Uniform scratches were made on the monolayers. The cells were treated with mitomycin C (10 μg/ml) to stop proliferation (51), and phase-contrast imaging of the cells at various time intervals (0, 24, 36, 48, and 60 h) was carried out using an Olympus IX71 microscope. The wounds were then analyzed by calculating the distance traveled. The distance of the wound closure was quantified by using ProgRes Capture Pro v2.8.8 (Olympus).

Transwell invasion was assayed using a modified Boyden chamber containing a polycarbonate Transwell membrane filter (8 μm pore size; Corning Costar, Cambridge, MA, USA) coated with collagen type I (38). Approximately 1,000 cells were seeded in the upper chamber in DMEM containing 1% FBS, and the invasion toward the lower chamber, containing DMEM with 10% FBS, after incubation for 24 (MDA-MB-231) or 48 (MCF7) hours at 37°C in 5% CO_2_ was measured. The invading cells remaining on the bottom surface of the membrane were stained with 0.5% crystal violet after scraping nonmigrated cells from the upper surface of the membrane, and the stained insert was washed and imaged.

### Microarray data analysis.

Microarray data for Star-PAP and PIPKIα knockdowns in HEK 293 cells were obtained from the NCBI Gene Expression Omnibus (GEO) (http://www.ncbi.nlm.nih.gov/geo/), accessible through GEO series accession number GSE9361 (17). We analyzed the data in line with the methods described previously (17) to obtain significant gene changes on the knockdowns and considered mRNAs with higher expression differences using a stringent threshold cutoff (*P* ≤ 0.05; fold change ≥ 2). Briefly, the data for Star-PAP and PIPKIα knockdown samples were compared with those for the control siRNA-treated samples, taken as baseline expression. Changes in expression were statistically analyzed using the empirical Bayes method implemented in the R package EBarrays, a publicly available statistical analysis system (http://www.r-project.org). We used the log-normal normal (LNN) expression model to calculate posterior probabilities of differential expression (DE). Of the >54,000 transcripts and variants, we identified ∼6,060 DE genes with a threshold of 0.888 to control the conditional false-discovery rate (cFDR) at 0.01 (*P* ≤ 0.05) for the Star-PAP knockdown and ∼6,186 DE genes with a threshold of 0.878 to control cFDR at 0.01 (*P* ≤ 0.05) using the LNN model. The fold changes of the intensity signals were calculated in Microsoft Excel as described previously (17). The genes were then sorted by their fold change values with a cutoff of less than or equal to 2-fold upregulation and less than or equal to −2-fold downregulation on the knockdowns to determine the genes significantly regulated by Star-PAP and PIPKIα.

## Supplementary Material

Supplemental material

## References

[1] Millevoi S, Vagner S 2010 Molecular mechanisms of eukaryotic pre-mRNA 3′ end processing regulation. Nucleic Acids Res 38:2757–2774. doi:10.1093/nar/gkp1176. 20044349 PMC2874999

[2] Wahle E, Ruegsegger U 1999 3′-end processing of pre-mRNA in eukaryotes. FEMS Microbiol Rev 23:277–295. 10371034 10.1111/j.1574-6976.1999.tb00400.x

[3] Zhao J, Hyman L, Moore C 1999 Formation of mRNA 3′ ends in eukaryotes: mechanism, regulation, and interrelationships with other steps in mRNA synthesis. Microbiol Mol Biol Rev 63:405–445. 10357856 10.1128/mmbr.63.2.405-445.1999 PMC98971

[4] Colgan DF, Manley JL 1997 Mechanism and regulation of mRNA polyadenylation. Genes Dev 11:2755–2766. doi:10.1101/gad.11.21.2755. 9353246

[5] Laishram RS 2014 Poly(A) polymerase (PAP) diversity in gene expression: star-PAP vs canonical PAP. FEBS Lett 588:2185–2197. doi:10.1016/j.febslet.2014.05.029. 24873880 PMC6309179

[6] Danckwardt S, Hentze MW, Kulozik AE 2008 3′ end mRNA processing: molecular mechanisms and implications for health and disease. EMBO J 27:482–498. doi:10.1038/sj.emboj.7601932. 18256699 PMC2241648

[7] Scorilas A 2002 Polyadenylate polymerase (PAP) and 3′ end pre-mRNA processing: function, assays, and association with disease. Crit Rev Clin Lab Sci 39:193–224. doi:10.1080/10408360290795510. 12120781

[8] Audic Y, Hartley RS 2004 Post-transcriptional regulation in cancer. Biol Cell 96:479–498. doi:10.1016/j.biolcel.2004.05.002. 15380615

[9] Lopez de Silanes I, Quesada MP, Esteller M 2007 Aberrant regulation of messenger RNA 3′-untranslated region in human cancer. Cell Oncol 29:1–17. 10.1155/2007/586139 PMC4618221 17429137

[10] Mayr C, Bartel DP 2009 Widespread shortening of 3′UTRs by alternative cleavage and polyadenylation activates oncogenes in cancer cells. Cell 138:673–684. doi:10.1016/j.cell.2009.06.016. 19703394 PMC2819821

[11] Sager R 1997 Expression genetics in cancer: shifting the focus from DNA to RNA. Proc Natl Acad Sci U S A 94:952–955. doi:10.1073/pnas.94.3.952. 9023363 PMC19620

[12] Chambers AF, Groom AC, MacDonald IC 2002 Metastasis: dissemination and growth of cancer cells in metastatic sites. Nat Rev Cancer 2:563–572. doi:10.1038/nrc865. 12154349

[13] Friedl P, Wolf K 2003 Tumour-cell invasion and migration: diversity and escape mechanisms. Nat Rev Cancer 3:362–374. doi:10.1038/nrc1075. 12724734

[14] Ridley AJ, Schwartz MA, Burridge K, Firtel RA, Ginsberg MH, Borisy G, Parsons JT, Horwitz AR 2003 Cell migration: integrating signals from front to back. Science 302:1704–1709. doi:10.1126/science.1092053. 14657486

[15] Bozzuto G, Ruggieri P, Molinari A 2010 Molecular aspects of tumor cell migration and invasion. Ann Ist Super Sanita 46:66–80. doi:10.4415/ANN_10_01_09. 20348621

[16] Mandel CR, Bai Y, Tong L 2008 Protein factors in pre-mRNA 3′-end processing. Cell Mol Life Sci 65:1099–1122. doi:10.1007/s00018-007-7474-3. 18158581 PMC2742908

[17] Mellman DL, Gonzales ML, Song C, Barlow CA, Wang P, Kendziorski C, Anderson RA 2008 A PtdIns4,5P2-regulated nuclear poly(A) polymerase controls expression of select mRNAs. Nature 451:1013–1017. doi:10.1038/nature06666. 18288197

[18] Li W, Anderson RA 2014 Star-PAP controls HPV E6 regulation of p53 and sensitizes cells to VP-16. Oncogene 33:928–932. doi:10.1038/onc.2013.14. 23416977 PMC3927653

[19] Li W, Laishram RS, Ji Z, Barlow CA, Tian B, Anderson RA 2012 Star-PAP control of BIK expression and apoptosis is regulated by nuclear PIPKIalpha and PKCdelta signaling. Mol Cell 45:25–37. doi:10.1016/j.molcel.2011.11.017. 22244330 PMC3268557

[20] Mohan N, Sudheesh AP, Francis N, Anderson R, Laishram RS 2015 Phosphorylation regulates the Star-PAP-PIPKIalpha interaction and directs specificity toward mRNA targets. Nucleic Acids Res 43:7005–7020. doi:10.1093/nar/gkv676. 26138484 PMC4538844

[21] Kandala DT, Mohan N, Vivekanand A, Sudheesh AP, Reshmi G, Laishram RS 2016 CstF-64 and 3′-UTR cis-element determine Star-PAP specificity for target mRNA selection by excluding PAPalpha. Nucleic Acids Res 44:811–823. doi:10.1093/nar/gkv1074. 26496945 PMC4737136

[22] Laishram RS, Barlow CA, Anderson RA 2011 CKI isoforms alpha and epsilon regulate Star-PAP target messages by controlling Star-PAP poly(A) polymerase activity and phosphoinositide stimulation. Nucleic Acids Res 39:7961–7973. doi:10.1093/nar/gkr549. 21729869 PMC3185439

[23] Laishram RS, Anderson RA 2010 The poly A polymerase Star-PAP controls 3′-end cleavage by promoting CPSF interaction and specificity toward the pre-mRNA. EMBO J 29:4132–4145. doi:10.1038/emboj.2010.287. 21102410 PMC3018792

[24] Gonzales ML, Mellman DL, Anderson RA 2008 CKIalpha is associated with and phosphorylates star-PAP and is also required for expression of select star-PAP target messenger RNAs. J Biol Chem 283:12665–12673. doi:10.1074/jbc.M800656200. 18305108 PMC2431003

[25] Barlow CA, Laishram RS, Anderson RA 2010 Nuclear phosphoinositides: a signaling enigma wrapped in a compartmental conundrum. Trends Cell Biol 20:25–35. doi:10.1016/j.tcb.2009.09.009. 19846310 PMC2818233

[26] Boronenkov IV, Loijens JC, Umeda M, Anderson RA 1998 Phosphoinositide signaling pathways in nuclei are associated with nuclear speckles containing pre-mRNA processing factors. Mol Biol Cell 9:3547–3560. doi:10.1091/mbc.9.12.3547. 9843587 PMC25675

[27] Bunce MW, Bergendahl K, Anderson RA 2006 Nuclear PI(4,5)P(2): a new place for an old signal. Biochim Biophys Acta 1761:560–569. doi:10.1016/j.bbalip.2006.03.002. 16750654

[28] Irvine RF 2003 Nuclear lipid signalling. Nat Rev Mol Cell Biol 4:349–360. doi:10.1038/nrm1100. 12728269

[29] Anderson RA, Boronenkov IV, Doughman SD, Kunz J, Loijens JC 1999 Phosphatidylinositol phosphate kinases, a multifaceted family of signaling enzymes. J Biol Chem 274:9907–9910. doi:10.1074/jbc.274.15.9907. 10187762

[30] Doughman RL, Firestone AJ, Anderson RA 2003 Phosphatidylinositol phosphate kinases put PI4,5P(2) in its place. J Membr Biol 194:77–89. doi:10.1007/s00232-003-2027-7. 14502432

[31] Spector DL, Lamond AI 2011 Nuclear speckles. Cold Spring Harbor Perspect Biol 3:a000646. doi:10.1101/cshperspect.a000646. PMC3039535 20926517

[32] Brader S, Eccles SA 2004 Phosphoinositide 3-kinase signalling pathways in tumor progression, invasion and angiogenesis. Tumori 90:2–8. 15143962 10.1177/030089160409000102

[33] Di Paolo G, De Camilli P 2006 Phosphoinositides in cell regulation and membrane dynamics. Nature 443:651–657. doi:10.1038/nature05185. 17035995

[34] Stafford LJ, Vaidya KS, Welch DR 2008 Metastasis suppressors genes in cancer. Int J Biochem Cell Biol 40:874–891. doi:10.1016/j.biocel.2007.12.016. 18280770

[35] Rubin EM, Guo Y, Tu K, Xie J, Zi X, Hoang BH 2010 Wnt inhibitory factor 1 decreases tumorigenesis and metastasis in osteosarcoma. Mol Cancer Ther 9:731–741. doi:10.1158/1535-7163.MCT-09-0147. 20197388 PMC2837364

[36] Yan J, Yang Q, Huang Q 2013 Metastasis suppressor genes. Histol Histopathol 28:285–292. doi:10.14670/HH-28.285. 23348381 PMC3910084

[37] Agarwal R, D'Souza T, Morin PJ 2005 Claudin-3 and claudin-4 expression in ovarian epithelial cells enhances invasion and is associated with increased matrix metalloproteinase-2 activity. Cancer Res 65:7378–7385. doi:10.1158/0008-5472.CAN-05-1036. 16103090

[38] Albini A 1998 Tumor and endothelial cell invasion of basement membranes. The matrigel chemoinvasion assay as a tool for dissecting molecular mechanisms. Pathol Oncol Res 4:230–241. 9761943 10.1007/BF02905254

[39] Weeraratna AT, Jiang Y, Hostetter G, Rosenblatt K, Duray P, Bittner M, Trent JM 2002 Wnt5a signaling directly affects cell motility and invasion of metastatic melanoma. Cancer Cell 1:279–288. doi:10.1016/S1535-6108(02)00045-4. 12086864

[40] Ferreira JP, Peacock RW, Lawhorn IE, Wang CL 2011 Modulating ectopic gene expression levels by using retroviral vectors equipped with synthetic promoters. Syst Synth Biol 5:131–138. doi:10.1007/s11693-011-9089-0. 23205156 PMC3234317

[41] Liu P, Cheng H, Roberts TM, Zhao JJ 2009 Targeting the phosphoinositide 3-kinase pathway in cancer. Nat Rev Drug Discov 8:627–644. doi:10.1038/nrd2926. 19644473 PMC3142564

[42] Thorpe LM, Yuzugullu H, Zhao JJ 2015 PI3K in cancer: divergent roles of isoforms, modes of activation and therapeutic targeting. Nat Rev Cancer 15:7–24. doi:10.1038/nrc3860. 25533673 PMC4384662

[43] Vivanco I, Sawyers CL 2002 The phosphatidylinositol 3-Kinase AKT pathway in human cancer. Nat Rev Cancer 2:489–501. doi:10.1038/nrc839. 12094235

[44] Thapa N, Tan X, Choi S, Lambert PF, Rapraeger AC, Anderson RA 2016 The hidden conundrum of phosphoinositide signaling in cancer. Trends Cancer 2:378–390. doi:10.1016/j.trecan.2016.05.009. 27819060 PMC5094282

[45] Ling K, Schill NJ, Wagoner MP, Sun Y, Anderson RA 2006 Movin' on up: the role of PtdIns(4,5)P(2) in cell migration. Trends Cell Biol 16:276–284. doi:10.1016/j.tcb.2006.03.007. 16616849

[46] Yu C, Gong Y, Zhou H, Wang M, Kong L, Liu J, An T, Zhu H, Li Y 2017 Star-PAP, a poly(A) polymerase, functions as a tumor suppressor in an orthotopic human breast cancer model. Cell Death Dis 8:e2582. doi:10.1038/cddis.2016.199. 28151486 PMC5386448

[47] Moffat J, Grueneberg DA, Yang X, Kim SY, Kloepfer AM, Hinkle G, Piqani B, Eisenhaure TM, Luo B, Grenier JK, Carpenter AE, Foo SY, Stewart SA, Stockwell BR, Hacohen N, Hahn WC, Lander ES, Sabatini DM, Root DE 2006 A lentiviral RNAi library for human and mouse genes applied to an arrayed viral high-content screen. Cell 124:1283–1298. doi:10.1016/j.cell.2006.01.040. 16564017

[48] Kingston RE, Chen CA, Rose JK 2003 Calcium phosphate transfection. Curr Protoc Mol Biol Chapter 9:Unit 9.1. doi:10.1002/0471142727.mb0901s63. 18265332

[49] Perocchi F, Gohil VM, Girgis HS, Bao XR, McCombs JE, Palmer AE, Mootha VK 2010 MICU1 encodes a mitochondrial EF hand protein required for Ca(2+) uptake. Nature 467:291–296. doi:10.1038/nature09358. 20693986 PMC2977980

[50] Gilbert C, Kristjuhan A, Winkler GS, Svejstrup JQ 2004 Elongator interactions with nascent mRNA revealed by RNA immunoprecipitation. Mol Cell 14:457–464. doi:10.1016/S1097-2765(04)00239-4. 15149595

[51] Li W, Fan J, Chen M, Guan S, Sawcer D, Bokoch GM, Woodley DT 2004 Mechanism of human dermal fibroblast migration driven by type I collagen and platelet-derived growth factor-BB. Mol Biol Cell 15:294–309. 14595114 10.1091/mbc.E03-05-0352 PMC307548

[52] Pfaffl MW 2001 A new mathematical model for relative quantification in real-time RT-PCR. Nucleic Acids Res 29:e45. 11328886 10.1093/nar/29.9.e45 PMC55695

