# Supplementary material for: Nuclear Phosphatidylinositol-Phosphate Type I Kinase α-Coupled Star-PAP Polyadenylation Regulates Cell Invasion
Source: Mol Cell Biol. 2018 Feb 12;38(5):e00457-17. doi: 10.1128/MCB.00457-17 (PMC5809686; doi:10.1128/MCB.00457-17)
Supplement: Supplemental material [file supp_38_5_e00457-17__index.html]

Supplemental material 

# Nuclear Phosphatidylinositol-Phosphate Type I Kinase α-Coupled Star-PAP Polyadenylation Regulates Cell Invasion

## Supplemental material

- Supplemental file 1 -

  Supplemental text and Fig. S1 (Measurement of uncleaved pre-mRNA levels), S2 (Wound healing assay in HeLa cells after knockdown of Star-PAP and PIPKIα), S3 (Sequence of CMV promoter), and S4 (Quantification of actual wound width)

  PDF, 8.2M
- Supplemental file 2 -

  Table S1 (List of common genes significantly regulated on Star-PAP and PIPKIα knockdown)

  XLSX, 134K
