# Supplementary material for: Nuclear Phosphatidylinositol-Phosphate Type I Kinase α-Coupled Star-PAP Polyadenylation Regulates Cell Invasion
Source: Mol Cell Biol. 2018 Feb 12;38(5):e00457-17. doi: 10.1128/MCB.00457-17 (PMC5809686; doi:10.1128/MCB.00457-17)
Supplement: Supplemental material [file MCB.00457-17_zmb999101701s1.pdf]

Supplementary data

Title

Nuclear phosphatidyl-inositol-phosphate type I kinase α coupled Star-PAP polyadenylation regulates cell invasion

Sudheesh AP and Rakesh S. Laishram[#]

Cardiovascular and Diabetes Biology Group, Rajiv Gandhi Centre for Biotechnology, Trivandrum - 014, India

[#] *Corresponding Author*

Running Title: Role of poly(A) polymerase Star-PAP in cancer cell invasion

[#]Corresponding Address:

Rakesh S. Laishram

Rajiv Gandhi Centre for Biotechnology

Thycaud Post, Poojappura

Trivandrum – 695014, India

E mail: laishram@rgcb.res.in

Phone: +91-0471-2529592

DNA Constructs and Antibodies

FLAG-tagged Star-PAP, FLAG-tagged PIPKIα, phosphodeficient S6A and ΔZF Star-PAP constructs were as described earlier(20). Different Star-PAP expressing mutants were constructed by site directed mutagenesis in the CMV promoter region (CAAT and TAATA box) of FLAG-Star-PAP construct in pCMV Tag 2A vector(40). Primers used for the site directed mutagenesis are shown in supplementary text. A total of 2 mutant constructs were generated in CAAT box at -62 and -61 positions, and 3 constructs in TAATA box at -29, -27, and -25 positions w.r.t. +1 transcription start site. A lentiviral vector system pLKO.1-TRC was modified and cloned to express the shRNA specific for Star-PAP and PIPKIα under U6 promoter in *AgeI/EcoRI* cloning sites of the vector as described earlier(47).

Rabbit polyclonal anti-Star-PAP(20), Rabbit polyclonal anti-PIPKIα(20), Rabbit polyclonal anti-CKIα (Bethyl lab), Rabbit monoclonal anti-β-Tubulin (Santacruz), Mouse monoclonal anti-FLAG M2 (Sigma), Rabbit monoclonal anti-FLAG (Sigma), Rabbit polyclonal anti-RNA polymerase II (Bethyl lab), Rabbit polyclonal anti-KISS1R (Abcam), Mouse monoclonal anti-E-Cadherin (Abcam), and Rabbit polyclonal anti-NME23A (Abcam), Goat polyclonal anti-NBK (Santacruz), Rabbit polyclonal anti-CPSF1 (Bethyl Lab) and Rabbit polyclonal anti-FOXA2 (Abcam), Mouse monoclonal anti-Neomycin phosphotransferase 2 (Abcam) were used for immunoblotting or RIP experiments as indicated.

Primers for Site directed mutagenesis

1. Star-PAP S6A F: 5′- TGGCGGCGGTGGATGCGGATGTCGAATCGCT -3′

   Star-PAP S6A R: 5′- AGCGATTCGACATCCGCATCCACCGCCGCCA -3′

2. Star-PAP Y573F F: 5′- GCAGCAGCCAATTTCTGCCGAAGCCT -3′

   Star-PAP Y573F R: 5′- AGGCTTCGGCAGAAATTGGCTGCTGC -3′

3. pCMVPromoter -62[2] F 5′- CGCCCCATTGACGGGAATGGGCGGTAGGC -3′

   pCMVPromoter -62[2] R 5′- GCCTACCGCCCATTCCCGTCAATGGGGCG -3′

4. pCMVPromoter -61[4] F 5′- GCCCCATTGACGCCCCCGGGCGGTAGGCG -3′

   pCMVPromoter -61[4] R 5′- CGCCTACCGCCCGGGGGCGTCAATGGGGC -3′

5. pCMVPromoter -29[2] F 5′- CGGTGGGAGGTCGGTATAAGCAGAGCTGG -3′

   pCMVPromoter -29[2] R 5′- CCAGCTCTGCTTATACCGACCTCCCACCG -3′

6. pCMVPromoter -25[3] F 5′- GTGGGAGGTCTATAGGGGCAGAGCTGGTTTA -3′

   pCMVPromoter -25[3] R 5′- TAAACCAGCTCTGCCCCTATAGACCTCCCAC-3′

7. pCMVPromoter -27[5] F 5′- GGTGGGAGGTCTAGGGGGGCAGAGCTGGTTTA -3′

   pCMVPromoter -27[5] R 5′- TAAACCAGCTCTGCCCCCCTAGACCTCCCACC -3′

Gene specific forward primers for 3′-RACE assay

1. CDH1 5′- GCCTGCTTTTGATGATGTCT -3′
2. CDH13 5′- AGGTCCACAGAGAGGGAGAG -3′
3. FEZ1 5′- GCATCTCTTCCCCAGAGAG -3′
4. KISS1R 5′- ACTGCTGGAACAGCGGCTA -3′
5. NME1 5′- ATTTGGAGGGAAGCTCTTGG -3′
6. WIF1 5′- TGTGGTAGTGGCATTTAAACAA -3′
7. GAPDH 5′- TTTGGCTACAGCAACAGGGT -3′

Primers for quantitative real time PCR

1. CDH1 FP: 5′- TGGGTTATTCCTCCCATCAG -3′

   RP: 5′- GTCACCTTCAGCCATCCTGT -3′

2. CDH13 FP: 5′- GCGGTATAATATCCGTCAGCA -3′

   RP: 5′- CCAGTCCAGCCATATCTTGAG -3′

3. FEZ1 FP: 5′- AACTTCCTCCCAGGCAGACT -3′

   RP: 5′- CTGCTGCACCAGCTCCTC -3′

4. KISS1R FP: 5′- GTGACCTTCCTCCTGTGCTG -3′

   RP: 5′- GGAACACCGTCACGTACCA -3′

5. WIF1 FP: 5′- CCTGGATAAAGGCATCATGG -3′

   RP: 5′- GAGAATGGTGTTGCCTTCAGA -3′

6. GCLC FP: 5′- AAGTTCTTGAAACTCTGCAAGAGAAGG -3’

   RP: 5′- GCCTCAACTGTATTGAACTCGGAC -3′

7. GAPDH FP: 5′- GAAGGTCGGAGTC AACGGATTT -3′

   RP: 5′- GAATTTGCCATGGGTGGAAT -3′

Primers for RIP analysis

1. GAPDH FP: 5′- CACACTGAATCTCCCCTCCT -3′

   RP: 5′- TTGACACAAGCCCAGCTTC -3′

2. CDH1 FP: 5′- TATTTTCTTTGGGGGTGGAA -3′

   RP: 5′- GCCTCTTTCTCCACGTTTTG -3′

3. KISS1R FP: 5′- TTGGTCTCTTGTGACGTTCG -3′

   RP: 5′- GGAATGATCCAGAAAGTCCTGT -3′

Primers for 3′-end cleavage

1. CDH1 ClvFP: 5′- TATTTTCTTTGGGGGTGGAA -3′

   ClvRP: 5′- GCCTCTTTCTCCACGTTTTG -3′
2. CDH13 ClvFP: 5′- TGTTTGCACTTGTGCTTTCAG -3′

   ClvRP- 5′- TCCAGCAGTTTTAACCACCAA -3′
3. FEZ1 ClvFP: 5′- CTGGCTACAGCCCTCAAAAA -3′

   ClvRP: 5′- AGAATGCTATACAGCCCGTCTC -3′
4. KISS1R ClvFP: 5′- TTGGTCTCTTGTGACGTTCG -3′

   ClvRP: 5′- GGAATGATCCAGAAAGTCCTGT -3′
5. WIF1 ClvFP: 5′- GAACTTTTTGCATTGGCTTGA -3′

   ClvRP: 5′- GTGCAAAGATCACCTCCACA -3′
6. GAPDH ClvFP: 5′- CACACTGAATCTCCCCTCCT -3′

   ClvRP: 5′- TTGACACAAGCCCAGCTTC -3′
7. GCLC ClvFP: 5′- ATGCCTGGTTTTCGTTTGCA -3′

   ClvRP: 5′- AGCTGTGGAACTCACACACACTCA -3′

siRNA Oligos

1. Control scrambled non-targeting : AGGUAGUGUAAUCGCCUUG
2. Star-PAP : GUGUGUUUGUCAGUGGCUU
3. PIPKIα : GAAGUUGGAGCACUCUUGG
4. CKIα : ON-TARGET SMART pool (Dharmacon)
5. FOXA2 : ON-TARGETplus SMART pool (Dharmacon)
6. CPSF-160 : ON-TARGET SMART pool (Dharmacon)

shRNAs

1. Star-PAP : GTGTGTTTGTCAGTGGCTT (Sense)
2. PIPKIα : GAAGTTGGAGCACTCTTGGTT (Sense)

Chemical Treatments

Cells were treated with Actinomycin D (2.5 µg/mL in DMSO, Sigma) and with cordycepin (100 µM in DMSO, Sigma) for various time points and the cells were harvested respectively for total RNA isolation.

Supplementary Figure Legend

Fig. S1: (A) Measurement of uncleaved pre-mRNA levels expressed relative to total mRNA (B) after treatment with Actinomycin at various time points as indicated, Cordycepin (24 hours) and siRNA specific to FOXA2, CPSF-160. Quantification of immunoblots in Fig. 2A expressed relative to β-tubulin of (C) Star-PAP, (D) PIPKIα, and (E) KISS1R. (F) Wound healing assay in various cell lines (HEK 293, MCF7, HeLa and MDA-MB-231) as in Fig. 2A at various time points post-wounding cells. (G) Quantification of wound healing assay in Fig. 2H expressed relative to 0 hour time point post wounding. Plot of actual wound gap in µm is shown in Supplementary Fig. 4C. Error bar represents s.e.m. (n=3). (H) Measurement of wound closure of F in various cell lines (HEK 293, MCF7, HeLa and MDA-MB-231) at various time points post-wounding. Quantification is expressed relative to 0 hour time point. Error bar represents s.e.m., n=3. Plot of actual wound gap at various time points in µm is shown in Supplementary Fig. 4G.

Fig. S2: (A) Wound healing assay in HeLa cells after knockdown of Star-PAP and PIPKIα. Actual wound width measured at various time points post-wounding (0 to 60 hours) and relative closure is shown in B and C. (D) qRT-PCR analysis of Star-PAP targets from MDA-MB-231 cells after ectopic expression of Star-PAP. Error bar represents s.e.m. (n=3). (E-F) Transwell invasion assay in modified Boyden chamber using MCF7 and MDA-MB-231 cells as indicated. Number of cells invaded toward the lower chamber stained with crystal violet in MCF7 (48 hours post-incubation) and MDA-MB-231 (24 hour post-incubation) under conditions as indicated. Quantification is shown in G and H, error bar represents s.e.m. of n=3 independent experiments.

Fig. S3: (A) Sequence of CMV promoter showing CAAT box and TATATAA box and various mutations as in Fig. 3. (B-D) Quantification of immunoblots of KISS1R, NME1 and NPTII in Fig. 3G normalized to internal control β-tubulin and plotted as % expression relative to wild type (WT) Star-PAP. (E-L) Westerns blot analysis after respective knockdowns in various cell lines as indicated.

Fig. S4: Quantification of the actual wound width (μm) at 0 to 60 hours post-wounding from Fig. 2B, C, H, Fig. 4A, Fig. 3C, Fig. 4F, and Supplementary Fig. 1F respectively. Error bar represents s.e.m. (n=3) for all experiments.

Supplementary Figure 1

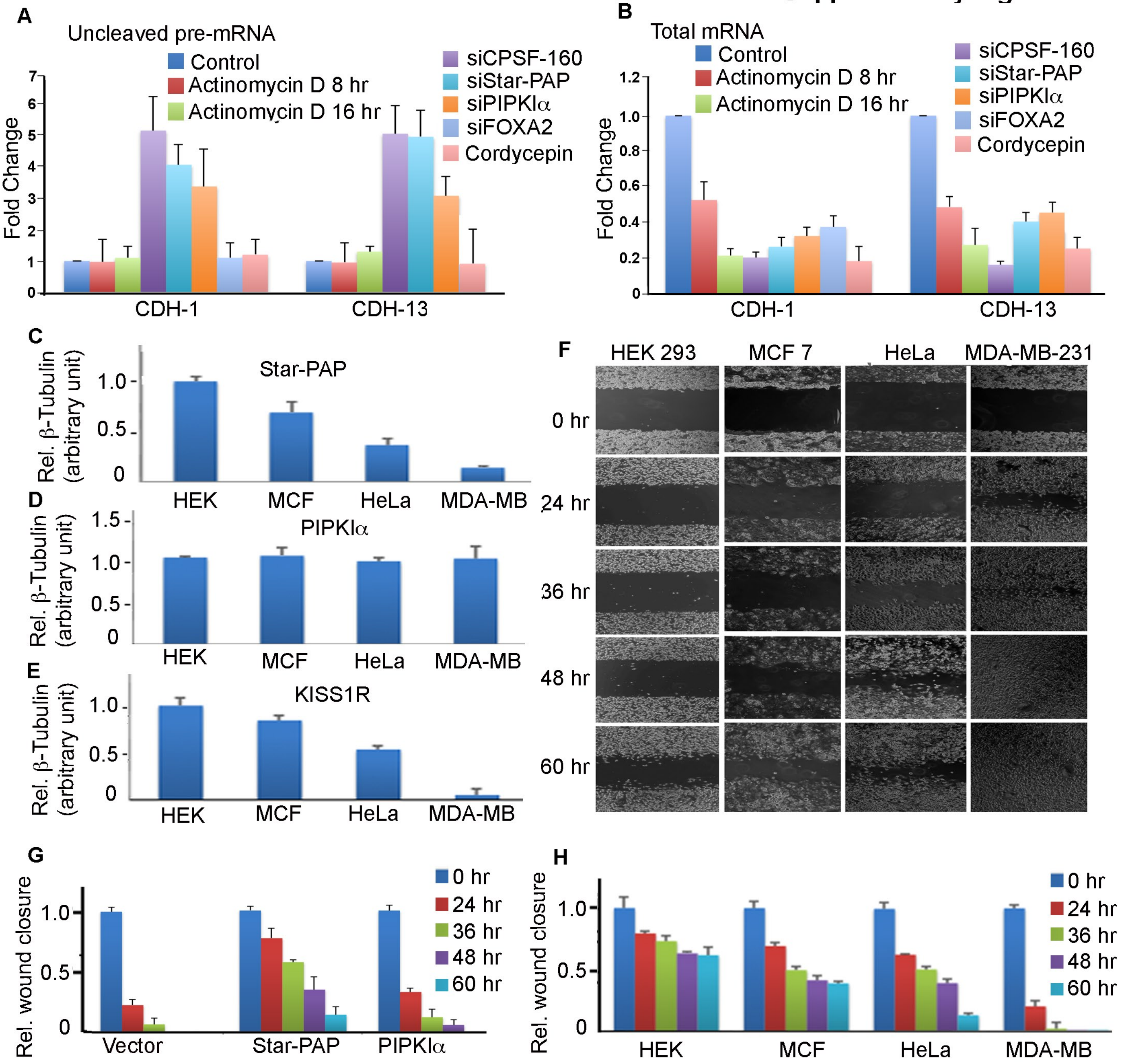

Supplementary Figure 2

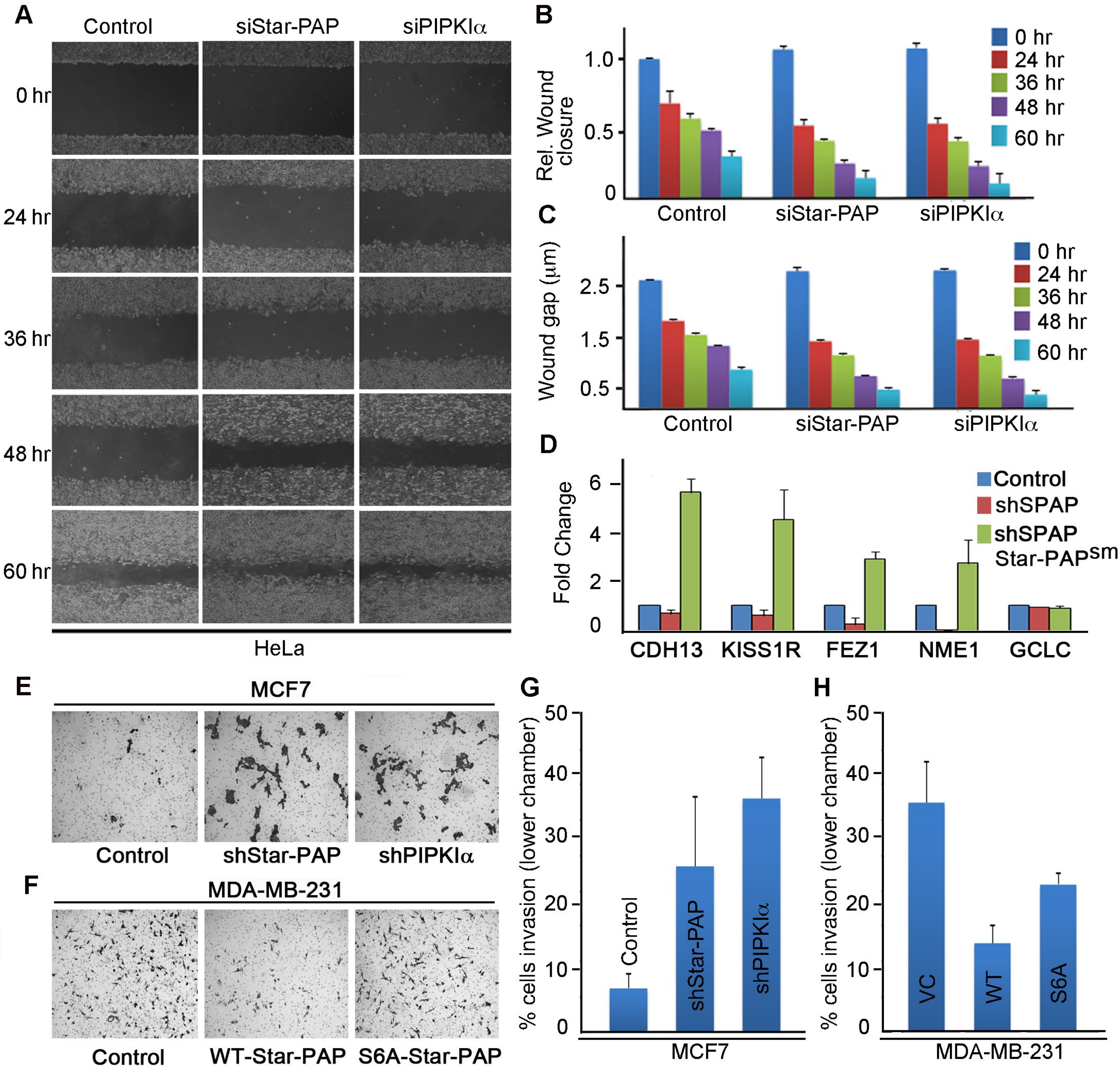

Supplementary Figure 3

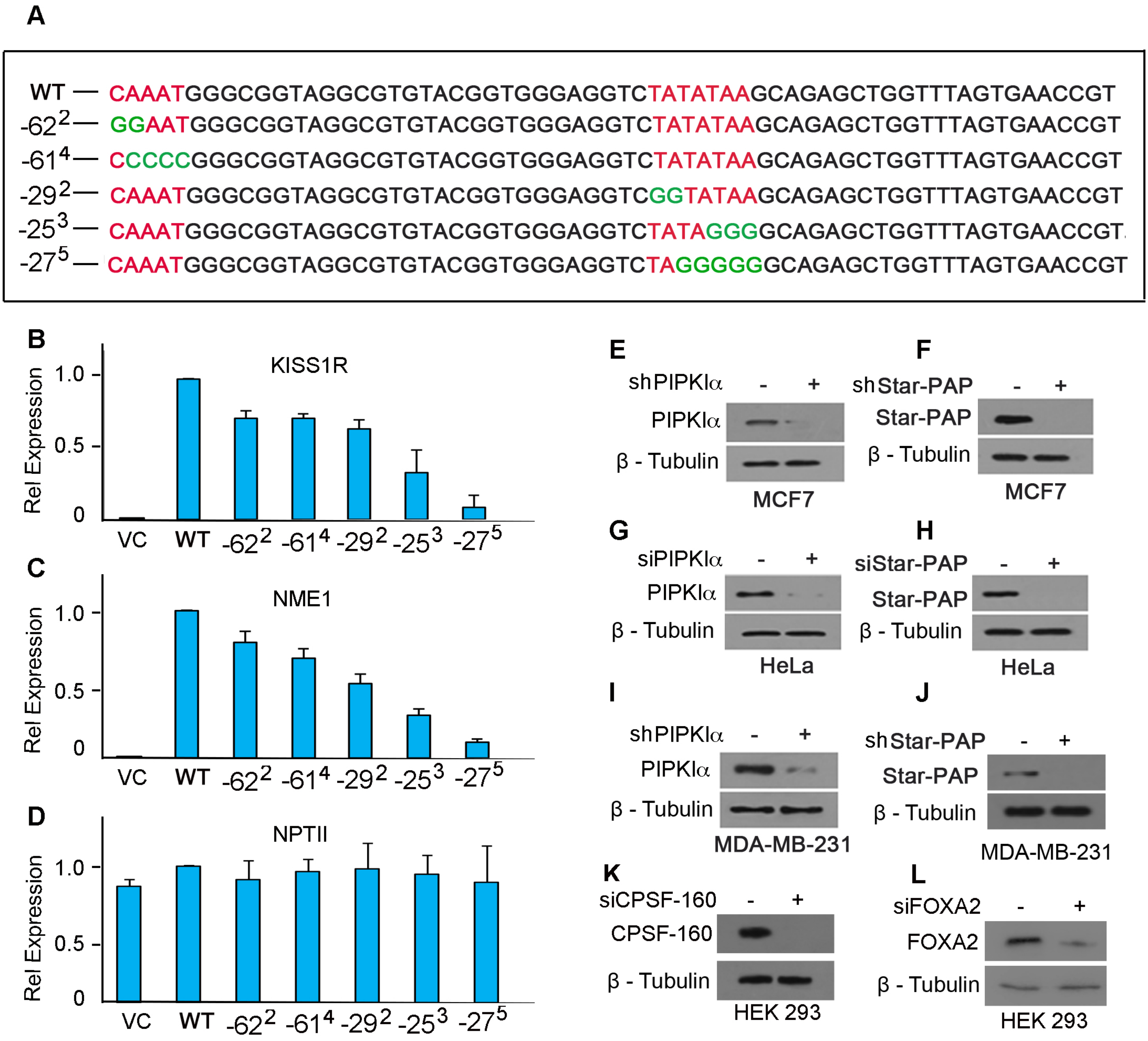

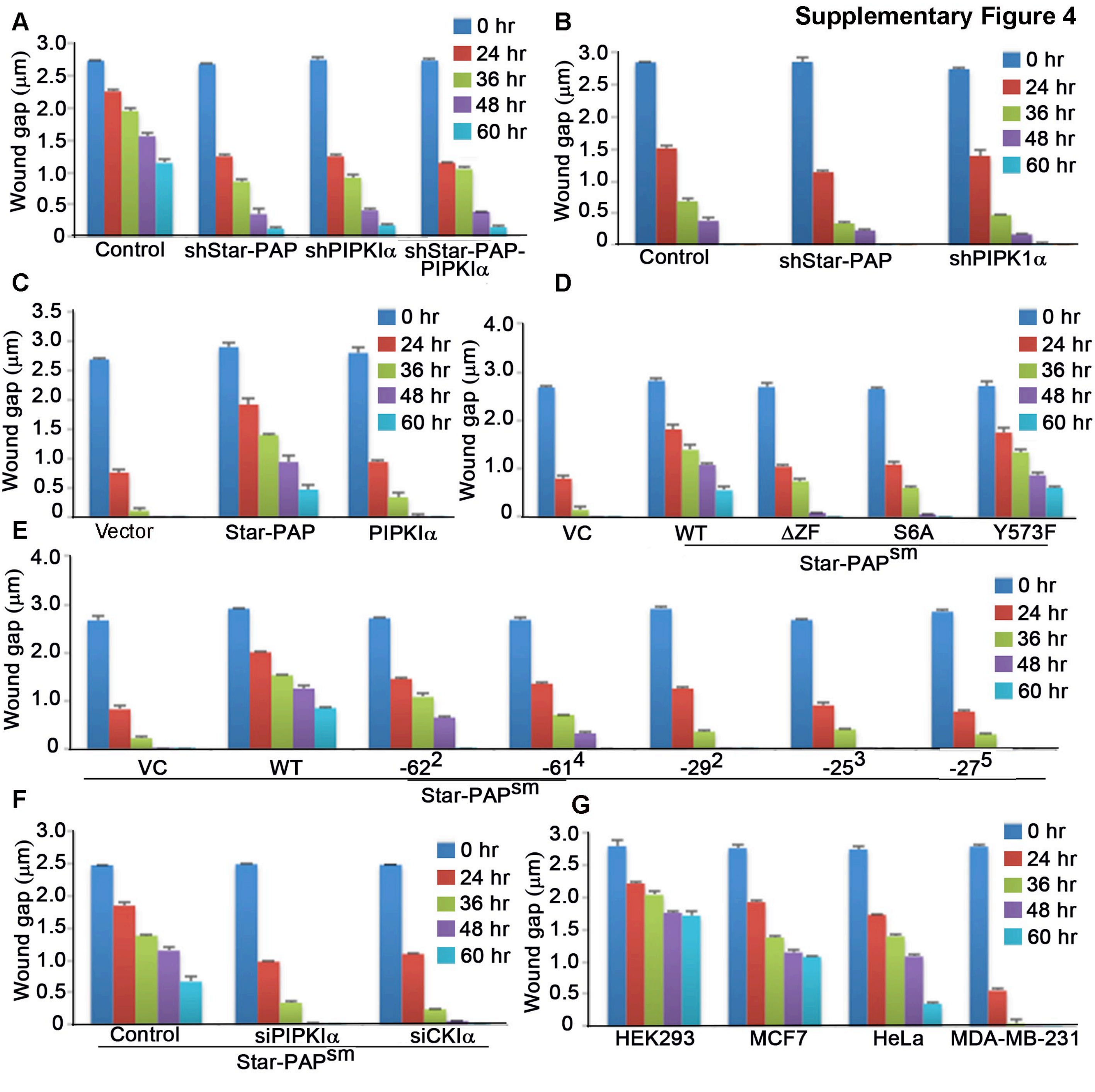
Supplementary Figure 4
A
Wound gap (μm)
0 hr
24 hr
36 hr
48 hr
60 hr
Control
shStar-PAP
shPIPKIα
shStar-PAP-PIPKIα
B
Wound gap (μm)
Control
shStar-PAP
shPIPK1α
C
Wound gap (μm)
Vector
Star-PAP
PIPKIα
D
Wound gap (μm)
VC
WT
ΔZF
S6A
Y573F
Star-PAPsm
E
Wound gap (μm)
VC
WT
-622
-614
-292
-253
-275
Star-PAPsm
F
Wound gap (μm)
Control
siPIPKIα
siCKIα
Star-PAPsm
G
Wound gap (μm)
HEK293
MCF7
HeLa
MDA-MB-231
